# Inhibitors of De Novo Guanylate Biosynthesis Enhance the Potency of MAPK Cascade Inhibitors Against Colorectal Cancer

**DOI:** 10.3390/ijms262411959

**Published:** 2025-12-11

**Authors:** Alexei A. Maslov, Nicholas H. Trageser, Julia V. Kichina, Haya Elamir, Evelyn Gardner, Frances Teaman, Vera Vishwanath, Scott M. Dugas, Anna Bianchi-Smiraglia, Katerina I. Leonova, Katerina V. Gurova, Mikhail A. Nikiforov, Eugene S. Kandel

**Affiliations:** 1 Department of Cell Stress Biology, Roswell Park Comprehensive Cancer Center, Buffalo, NY 14263, USA; 2 Department of Immunology, Roswell Park Comprehensive Cancer Center, Buffalo, NY 14263, USA; 3 Department of Pathology, Duke University, Durham, NC 27710, USA

**Keywords:** targeted therapy, colorectal carcinoma, mitogen-activated protein kinase

## Abstract

Despite continuing improvement in the standard of care, the clinical outcomes in metastatic colorectal cancer (CRC) remain poor, especially among patients whose tumors carry activating mutations in BRAF or RAS-family oncogenes. These mutations initiate a series of oncogenic signal transduction events, known as the mitogen-activated protein kinase (MAPK) cascade. While therapeutic targeting of this pathway achieved impressive results in other malignancies, the effectiveness of this approach remains low in CRC. In the current study, we observed that inhibitors of GTP production synergize with various inhibitors of the MAPK cascade in suppressing a variety of CRC cell lines. Furthermore, we discovered that an inhibitor of guanylate biosynthesis increases the efficacy of MAPK cascade inhibitors against human CRC grown in mice. Moreover, a combination of MEK and guanylate biosynthesis inhibitors is more potent than the MEK inhibitor alone in increasing the efficacy of immune therapy in an immunocompetent mouse model. Considering that guanylate biosynthesis inhibitors are already used in clinical practice for other applications, their use in synergistic combinations with the inhibitors of the MAPK cascade may present an actionable strategy to increase the efficacy of the latter.

## 1. Introduction

Excluding skin cancers, colorectal cancer (CRC) is the third most prevalent malignant tumor worldwide and the second most lethal one [1], with >150 K new cases and >52 K deaths yearly in the US alone [2]. Colonoscopy is effective in preventing CRC by removing precancerous lesions, while early excision offers cure rates of ~90% for localized disease [3]. Unfortunately, most cases are diagnosed at regional or distant stages [2], and the 5-year survival rate for the latter is below 15% [2]. In numerous trials of various regimens of conventional chemotherapy, typical objective response rates in metastatic CRC remained below 50%, while median overall survival under even the best regimens seldom exceeded 1 year, despite frequent and sometimes life-threatening side effects [4]. Unlike some other cancers, where immunotherapy generally availed a dramatically improved standard of care, the benefit of this treatment alone in CRC is restricted to 3–7% of patients with defective mismatch repair (MMR). In this group, immune therapy can achieve some durable responses, albeit in a minority of patients [5,6].

Hopes of improved outcomes were associated with the growing understanding of CRC molecular pathology. Inhibition of EGFR delivers tangible improvement to CRC care, but by itself is ineffective in RAS- or BRAF-mutated cases. Approximately 50% of metastatic CRCs are driven by mutant KRAS and NRAS [7], and ~10% by mutant BRAF [7,8]. These mutations lead to constitutive activation of the mitogen-activated protein kinase (MAPK) cascade, which includes sequential activation of MEK and ERK enzymes, with the latter being directly involved in the changes that stimulate proliferation, survival, invasion, immune evasion, and enhanced metabolism in cancer cells. In various treatment scenarios, mutations in KRAS, NRAS, and BRAF portend a poor prognosis in CRC [8,9]. While the RAS mutants most common in CRC are still undruggable by FDA-approved pharmacological agents, targeting BRAF and its downstream effector MEK gained encouraging results in melanoma [10,11]. Unfortunately, objective responses to BRAF and MEK inhibitors (BRAFi and MEKi) are very rare in CRC [12]. The likely explanation is the difficulty in attaining complete inhibition of the MAPK cascade, which can be reactivated by various upstream factors. As EGFR may be one such factor, various combinations of BRAF and EGFR inhibitors were tested, achieving objective responses in a minority of patients and only slight increases in progression-free or overall survival [13,14]. It was concluded that new approaches are needed to effectively inhibit the MAPK cascade [13], and a quantitative increase in the efficacy of MAPK suppression could make a major difference in the outcomes [15]. Recently, an improvement in the care of BRAF-mutant CRC was achieved by combining MEK and EGFR inhibitors with the mFOLFOX6 chemotherapy regimen, but about 40% of the cases still were non-responsive [16]. In RAS-mutant CRC, a wide adoption of RAS inhibitors is yet to come, but this strategy in other cancers already faces readily emerging drug resistance [17]. Moreover, the diversity of RAS mutations in CRC may necessitate the use of pan-RAS inhibitors, which are yet to be widely accepted for clinical practice. Thus, an effective therapy for RAS- and BRAF-driven metastatic CRC remains a major unmet public health need.

PAK1 (p21-activated protein kinase) and its eponymous activator RAC1 (also known as p21^RAC1^) are important components of signaling by oncogenic RAS proteins and represent a targetable vulnerability in cancer cells driven by RAS oncogenes [18,19,20,21,22,23,24]. PAK1 has been implicated in the co-stimulation of both CRAF and MEK1 [24,25,26,27], as well as in a variety of other oncogenic phenomena [23,24]. Prior work has revealed that PAK1 is essential for RAS-driven cancer cells, including those of CRC [20,22], and inhibition of PAK1 sensitizes RAS- and GNAQ-mutant cells to MAPK cascade inhibitors [22,28]. While cells with activating BRAF mutations are generally more resistant to PAK inhibition [22,29], the latter was shown to sensitize them to BRAF and MEK inhibitors in several cancer models [30,31]. In contrast, inhibition of PAK1 in non-transformed cells is much less toxic and may be cytoprotective in some conditions [32]. Unfortunately, direct inhibitors of RAC and PAK proteins have yet to be adopted into clinical practice. Interestingly, it transpired that RAC1 directly associates with the enzymes of GTP metabolism and is highly dependent on the locally produced GTP for its activity [33,34,35]. This high reliance on de novo synthesis results in significant downregulation of RAC1 activity in conditions that have only a modest effect on the overall abundance of GTP in the cell [33]. Importantly, various inhibitors of guanylate biosynthesis are already in clinical use. This led to the hypothesis that the efficacy of MAPK cascade inhibition may be improved by concomitant targeting of GTP biosynthesis [28,36]. In the present study, we set forth to test this hypothesis in the context of colorectal cancer.

## 2. Results

### 2.1. RAC and MEK Inhibitors Synergistically Suppress CRC Cells

**Figure 1:**
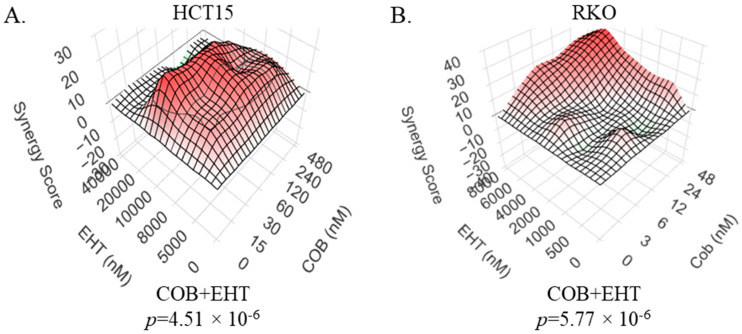
Inhibitors of MEK and RAC synergize in suppressing colorectal cancer cell lines. (**A**) HCT15 KRAS-mutant colorectal cancer cells were treated for 4 days with various doses of either the RAC inhibitor EHT-1864 (“EHT”) or the MEK inhibitor cobimetinib (“COB”), alone and in combinations. After comparing the numbers for remaining cells using methylene blue staining and extraction method, Loewe synergy plots were generated, along with the accompanying *p*-values, using SynergyFinder software, as described in the Materials and Methods. (**B**) RKO BRAF-mutant colorectal cancer cells were treated for 3 days with either EHT-1864 or cobimetinib, alone and in combinations. Synergy plots and associated *p*-values were generated as in (**A**).

RAC1 and its downstream effector PAK1 have emerged as mediators of therapeutic resistance and sensitivity in the context of various malignancies [28,30,37,38,39]. Considering that interference with PAK1 function makes at least some BRAF- and RAS-driven cancer cells more vulnerable to the inhibitors of the MAPK cascade [22,30,31], we tested whether inhibition of RAC would have a similar effect. Indeed, we observed that in colorectal cancer cell lines HCT15 (RAS-mutant) and RKO (BRAF-mutant), MEK and RAC inhibitors exhibit a synergistic suppressive activity (Figure 1). This further underscores the relevance of RAC-dependent signaling as a modulator of cell response to targeted therapy, as well as a target for improving the efficacy of the treatment.

### 2.2. IMPDH Inhibitors Synergize with the Inhibitors of the MAPK Cascade Against CRC Cells

While PAK and RAC inhibitors are still in preclinical development, an opportunity to control RAC signaling is suggested by the observation that RAC1, unlike some other GTPases, is highly vulnerable to even modest reduction in de novo GTP biosynthesis, and at least some of the consequences of modest GTP depletion can be reversed by expression of a hyperactive RAC1 mutant with reduced GTP-hydrolyzing activity [35]. GTP distribution in a cell is not random [40], and RAC1 activity coincides with higher local GTP availability [33], which is likely due to a direct association between RAC1 and the enzymes of de novo GTP biosynthesis, such as IMPDH [33]. Thus, it is conceivable that clinically available IMPDH inhibitors, even at relatively modest doses, could sensitize cells to MAPK cascade inhibitors by modulating the activity of the RAC/PAK signaling axis [28,36].

**Figure 2:**
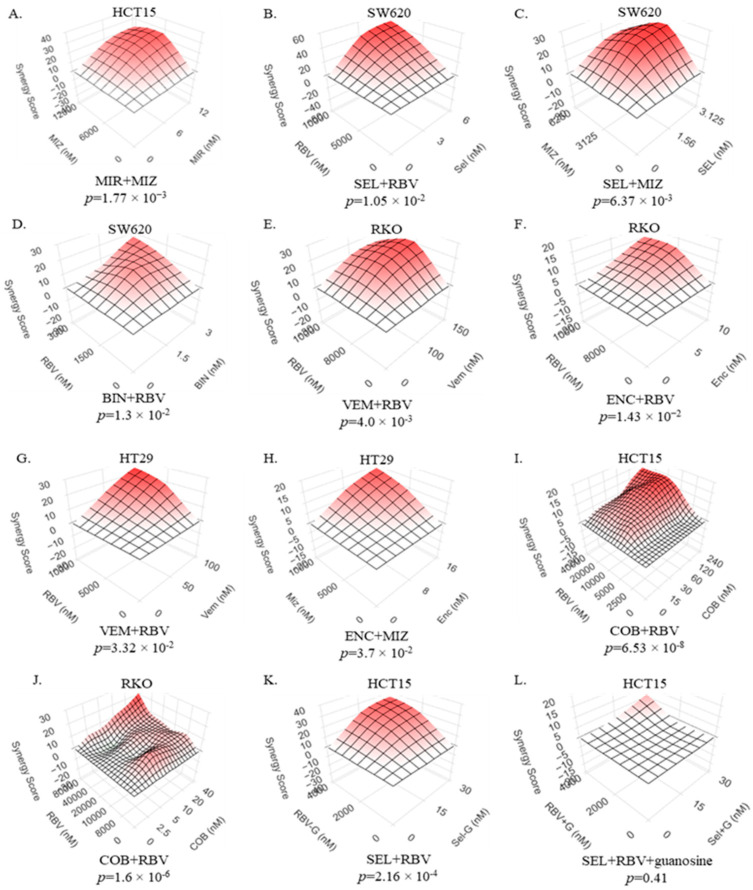
IMPDH inhibitors synergize with the inhibitors of the MAPK cascade in suppressing colorectal cancer cell lines. (**A**) HCT15 cells treated with mizoribine and mirdametinib. (**B**) SW620 cells treated with ribavirin and selumetinib. (**C**) SW620 cells treated with mizoribine and selumetinib. (**D**) SW620 cells treated with ribavirin and binimetinib. (**E**) RKO cells treated with ribavirin and vermurafenib. (**F**) RKO cells treated with ribavirin and encorafenib. (**G**) HT29 cells treated with ribavirin and vemurafenib. (**H**) HT29 cells treated with mizoribine and encorafenib. (**I**) HCT15 cells treated with ribavirin and cobimetinib. (**J**) RKO cells treated with ribavirin and cobimetinib. (**K**) HCT15 cells treated with ribavirin and selumetinib. (**L**) HCT15 cells treated with ribavirin and selumetinib in the presence of 25 µM guanosine. The data were collected, analyzed and presented as in Figure 1A. “MIZ”-mizoribine (IMPDH inhibitor). “MIR”-mirdametinib (MEK inhibitor). “SEL”—selumetinib (MEK inhibitor). “RBV”—ribavirin (IMPDH inhibitor). “BIN”—binimetinib (MEK inhibitor). “VEM”—vemurafenib (BRAF inhibitor). “ENC”—encorafenib (BRAF inhibitor). “COB”—cobimetinib (MEK inhibitor).

Predictably, the treatment of colorectal cancer cells with modest doses of IMPDH inhibitors ribavirin and mizoribine reduces the levels of GTP in cells (Figure S1). As was observed in other systems before [33,36], IMPDH inhibitors also reduced the activity of RAC1, as attested by reduced ability of RAC1 to bind a PAK1-based bait (Figure S2). Importantly, we observed that these IMPDH inhibitors synergize with the inhibitors of the MAPK cascade: various MEK inhibitors (cobimetinib, selumetinib, binimetinib, mirdametinib) and, in BRAF-mutant cells, BRAF inhibitors (encorafenib, vemurafenib) (Figure 2A–K). It is noteworthy that, despite differences in molecular structures and chemical properties of these compounds, the inhibitors of each enzyme display similar behaviors in the synergy studies, indicating that the phenomenon reflects their on-target activity. The importance of GTP biosynthesis in our system is further demonstrated by the fact that the synergy was completely abolished when cells were supplemented with guanosine (compare Figure 2K,L), which can be utilized by cells for the salvage pathway of GTP production [41].

**Figure 3:**
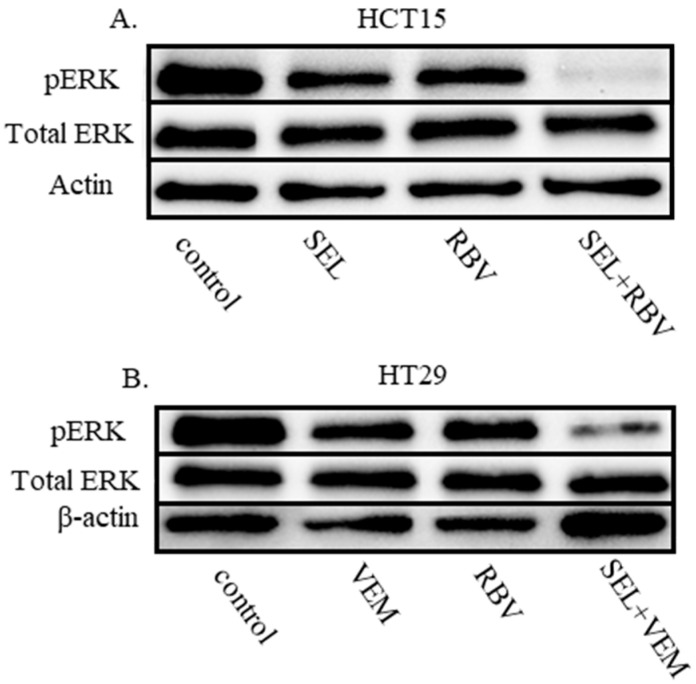
An IMPDH inhibitor improves suppression of the MAPK cascade by inhibitors of MEK and BRAF. (**A**) HCT15 cells were lysed after a 24-h treatment with 40 nM selumetinib (“SEL”), 4 uM ribavirin (“RBV”), a selumetinib-ribavirin combination, or the vehicle control (DMSO). The lysates were probed by immunoblotting for phosphorylated ERK (pERK), total ERK and β-actin (loading control). (**B**) HT29 cells were lysed after a 48-h treatment with 100 nM vemurafenib (“VEM”), 10 uM ribavirin (“RBV”), a vemurafenib-RBV combination, or the vehicle control (DMSO). The lysates were probed by immunoblotting for phosphorylated ERK (pERK), total ERK and β-actin (loading control).

ERK phosphorylation is an indicator of the activity of the MAPK cascade, which is often reduced by impacts that cooperate with MEK and BRAF inhibitors [30,42]. Accordingly, synergistic drug combinations of IMPDHi with MEKi and BRAFi resulted in a pronounced reduction in the levels of phospho-ERK even in the conditions when individual compounds showed only a modest effect (Figure 3). Importantly, the experience with targeted therapies in the clinics suggests that effective reduction in phospho-ERK levels signals a better therapeutic response, while the inability to reduce the phosphorylation and, hence, the activity of ERKs is often associated with reduced efficacy [13,43,44]. Thus, the more effective control of the MAPK cascade by the drug combination suggests a possibility for better tumor control in vivo.

### 2.3. An IMPDH Inhibitor Enhances the Potency of MEK Inhibitors In Vivo

**Figure 4:**
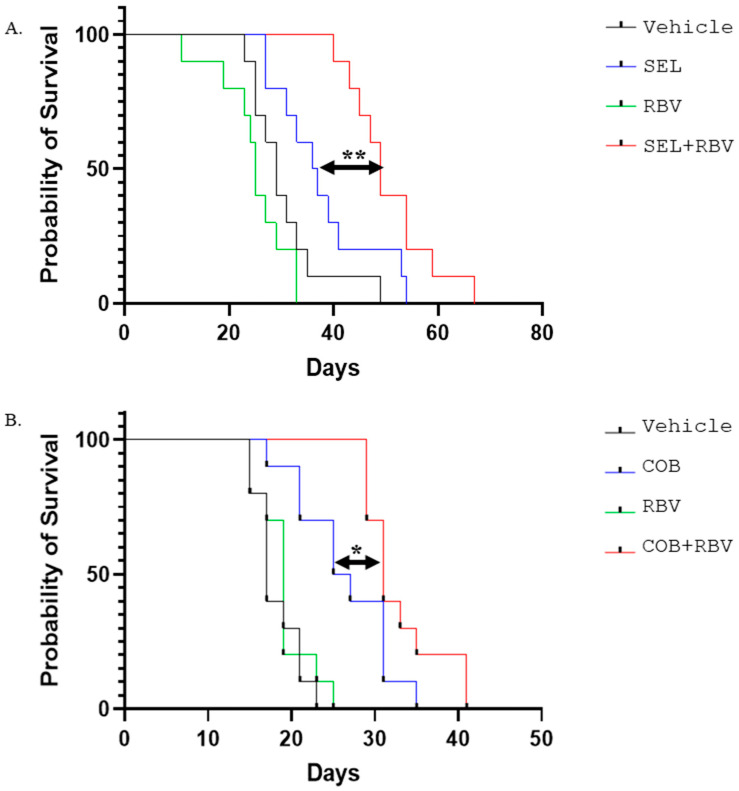
An IMPDH inhibitor increases the effect of MEK inhibitors in in vivo xenograft models. (**A**) HCT15 xenografts were established by injecting cells subcutaneously into the flanks of SCID mice. After tumors reached the volume of approximately 100 mm^3^, daily gavage treatments were started with 30 mg/kg selumetinib (“SEL”) and 20 mg/kg ribavirin (“RBV”), alone or in combination, or the respective vehicle control with 10 mice per group. Kaplan–Meier survival curves for each treatment group were prepared and analyzed using GraphPad Prism 9. ** *p* < 0.005. (**B**) RKO xenografts were established as in (A). After tumors reached approximately 100 mm^3^, daily gavage treatments were started with 5 mg/kg cobimetinib (“COB”) and 20 mg/kg ribavirin (“RBV”), alone or in combination, or the respective vehicle control with 10 mice per group. Survival analysis was done as in (A). * *p* < 0.05.

In order to explore the benefit of combining MEK and IMPDH inhibitors in the context of an organism, we treated mice harboring xenografts of RAS-mutant colorectal carcinoma HCT15 with MEK inhibitor selumetinib and IMPDH inhibitor ribavirin, singly or in combination (Figure 4A). At the dosage used, the effect of ribavirin alone was statistically indistinguishable from that of the vehicle control, while selumetinib alone offered a survival benefit. Importantly, this benefit was significantly increased by the addition of ribavirin (Figure 4A). Similarly, the addition of ribavirin enhanced the efficacy of MEK inhibitor cobimetinib against xenografts of BRAF-mutant colorectal carcinoma RKO, even though ribavirin alone had no discernible effect on survival (Figure 4B). These observations suggest that combinations of MEK and IMPDH inhibitors may have anti-cancer activity well above that of their individual components.

### 2.4. A Combination of IMPDH and MEK Inhibitors Enhances the Survival Benefits of Immune Checkpoint Inhibition in a Syngeneic Xenograft Model

**Figure 5:**
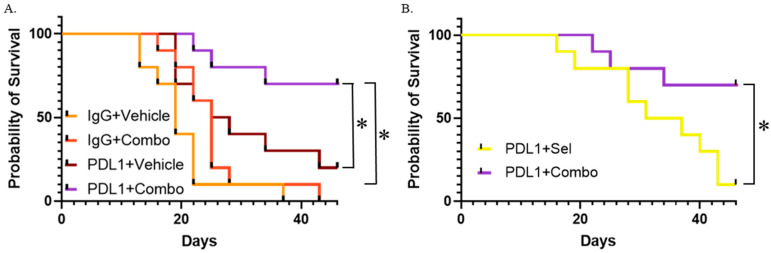
A combination of an IMPDH inhibitor and a MEK inhibitor enhances the effect of immune therapy in mice. On day 1 of the experiment, BALB/c mice bearing CT26 tumors (50 mm^3^–100 mm^3^) were randomly assigned into two groups to be injected interperitoneally with either 200 ug of anti-PDL1 antibody (an immune checkpoint inhibitor, ICI), or 200 ug of an IgG isotype control. The injections were carried 3 times (days 1, 4 and 7). On the 10th day of the experiment, the mice were randomly assigned to groups for daily gavage with the indicated agents. Kaplan-Meier survival plots were produced and analyzed using GraphPad Prism 9. (**A**) A targeted therapy combination (30 mg/kg selumetinib + 20 mg/kg ribavirin; “combo”) with an immunotherapy lead-in is more effective that the combo or the ICI alone. (**B**) A regimen containing the selumetinib + ribavirin combination targeted therapy is superior to the one containing 30 mg/kg selumetinib-only targeted therapy. * *p* < 0.05.

Immune therapy offers a well-documented survival benefit for colorectal carcinoma patients whose tumors are deficient in mismatch repair, and, in some cases, such an intervention is not only life-prolonging but actually curative [45,46]. While mismatch repair-proficient variants of certain malignancies are also responsive to immune therapies [47,48], the situation is unfortunately different in colorectal carcinoma [45]. MAPK cascade activity has been implicated in immune evasion in various models, and inhibition of this pathway was shown to enhance the efficacy of immune therapies in various preclinical models [49,50,51,52,53,54,55]. Considering our observations of the synergy between MEK and IMPDH inhibitors, as well as the fact that ribavirin RBV stimulates a pathway of T-cell differentiation [56,57] and activates DC cells [58,59] in a way favorable for anti-cancer activity, we tested the effect of this drug combination on the efficacy of an immune therapy. We used immune-competent BALB/c mice that carried well-established, rapidly growing xenografts of a syngeneic CT26 (KRAS-mutant) colorectal carcinoma. We used an immunotherapy lead-in, followed by targeted therapy. A follow-up with a targeted therapy was previously shown to improve the outcomes for immune checkpoint inhibitors (ICI) in preclinical models [49,50]. In our experiments, anti-PDL1 antibodies played the role of an ICI and were followed by a ribavirin/selumetinib combination (Figure 5A). The survival benefit of this combination by itself was not statistically significant when following pre-treatment with an inactive isotype control antibody. ICI alone offered a significant, albeit modest, increase in survival when followed by a vehicle control instead of a targeted therapy. Importantly, the survival benefit was greatly enhanced when the ICI treatment was followed by the combined targeted therapy. Notably, the drug combination was much more effective than selumetinib alone in enhancing the therapeutic effect of immunotherapy (Figure 5B). This indicates that ribavirin plays an important role in the synergy between the therapeutic modalities.

### 2.5. Ribavirin Synergizes with a Pan-RAS Inhibitor in Suppressing KRAS-Mutant CRC Cells

**Figure 6:**
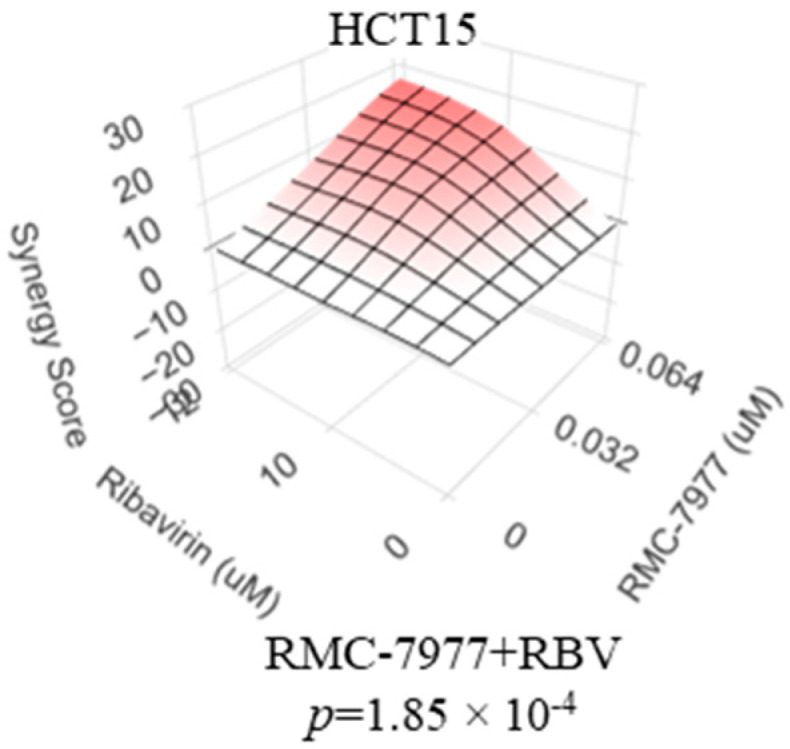
An inhibitor of IMPDH synergizes with a pan-RAS inhibitor in suppressing HCT15 colorectal cancer cells. HCT15 KRAS-mutant colorectal cancer cells were treated for 5 days with various doses of either the pan-RAS inhibitor RMC-7977 or the IMPDH inhibitor ribavirin, alone and in combinations. After comparing the numbers of the remaining cells using methylene blue staining and extraction method, a Loewe synergy plot was generated, along with the accompanying *p*-value, using SynergyFinder software, as described in the Materials and Methods.

Therapeutic agents targeting activated RAS proteins have recently emerged as a novel treatment option for cancers driven by these oncogenes. Inhibitors of activated KRAS-mutant KRAS^G12C^ proved to be beneficial in lung cancer, including heavily pretreated patients [60]. However, the frequency of this RAS variant in metastatic CRC is quite low, and the benefits of KRAS^G12C^ inhibitors, even among the carriers of this mutation, are considerably lower in CRC than in the lung cancer setting [61]. Among the avenues to improve the efficacy of this approach is to expand the breadth of targets to other mutant and, possibly, wildtype forms of RAS, as well as to identify interventions that synergize with this category of drugs. Encouragingly, we observed that ribavirin synergizes with a prototype pan-RAS inhibitor (RMC-7977 [62]) in suppressing KRAS^G13D^-driven HCT-15 cells (Figure 6). This early-stage observation, albeit limited in scope, supports further investigation into combinations of the emerging RAS inhibitors with those of IMPDH.

## 3. Discussion

The role of the RAC/PAK signaling axis in modulating the efficacy of conventional inhibitors of the MAPK cascade is well established in many different models, including CRC ([28], Figure 1). Safe and effective selective inhibitors of RAC/PAK signaling are yet to enter clinical practice. Notably, de novo GTP biosynthesis, and its key enzyme IMPDH2 in particular, plays an important role in the regulation of RAC activity, making RAC1 vulnerable to treatments that cause even a modest reduction in cellular GTP levels [33,35]. We thus tested the hypothesis that modest doses of IMPDH inhibitors would act cooperatively with the inhibitors of the MAPK cascade against CRC cells that have BRAF or RAS mutations as oncogenic drivers. The results of our experiments are in agreement with this hypothesis: synergistic suppression of various BRAF- and RAS-mutant CRC cell lines was achieved by various combinations of MEK and IMPDH inhibitors. Furthermore, in BRAF-mutant cells, IMPDH inhibitors synergize with those of BRAF. The fact that synergy is seen with multiple chemically distinct compounds designed to inhibit the same enzyme suggests the phenomenon is based on their on-target activity. The relevance of guanylate biosynthesis is further underscored by the fact that drug synergy can be abolished by guanosine supplementation (Figure 2K–L) and that previously characterized shRNAs against IMPDH2 [33,63] significantly reduce the IC50 values for inhibitors of MEK and BRAF in BRAF-mutant RKO cells (Figure S3).

The synergy in reducing cell numbers was paralleled by more effective reduction of phospho-ERK levels (Figure 3), a common measure of the MAPK cascade activity. Although this effect provides the most parsimonious explanation for the efficacy of the drug combinations, it remains possible that the drugs, individually or combined, affect other oncogenic properties of a cell. Importantly, the combinations remain active in the context of an organism, where they surpass individual drugs in extending the survival of mice bearing human CRC xenografts (Figure 4). Notably, IMPDH inhibitor ribavirin is already widely used in clinics as an anti-hepatitis C drug [64]. Ribavirin was proposed as a possible anti-cancer agent, but minimal activity (discussed in [65]) or failure [66] ensued, with no wide adoption in clinical oncology. Our observations suggest that the drug can augment the efficacy of other targeted therapies even at doses that are not efficacious as monotherapies.

Safety of the new drug combinations is a matter of great importance. The animals in our experiments were monitored daily for these signs of morbidity or distress: ruffled fur, weight loss over 20%, ocular discharge, lethargy, hunched back, inappetence, ataxia, tremors, diarrhea, huddled appearance, respiratory rate change (slow, shallow, labored, rapid), and lateral recumbency. For the whole duration of the reported experiments, none of these signs was observed in any of the animals treated with any combination of IMPDH and MEK inhibitors. This agrees with the observation in a recent study, where the selumetinib and ribavirin combination was administered to mice for over 100 days [36]. Some published reports describe the successful administration of ribavirin to mice at doses as high as 300 mg/kg [67]. Clinical relevance of such a dose, which is 15 times higher than the dose used in our study, is uncertain. In contrast, based on the comparison of the reported plasma concentrations of ribavirin in patients and in variously treated mice [68,69,70], the dosage of this drug in our in vivo experiments is expected to resemble both what is clinically feasible in humans and what yields synergistic effects in vitro. Nevertheless, a much more thorough investigation of possible signs of damage to different tissues and organ systems is warranted in order to obtain a more definitive conclusion on possible side effects of the drug combinations. The safety considerations may also be key in determining which drug combination is better suited for clinical translation. While the distinct inhibitors that target the same enzyme acted similarly in our synergy assays, the differences in their safety profiles, along with pharmacokinetic and pharmacodynamic properties, would need to be considered for future applications. It is conceivable that different combinations will be better suited for different categories of patients.

While the combination of MEK and IMPDH inhibitors significantly prolonged the survival of mice xenografted with human CRC cells (Figure 4), we believe that there may be ways to increase this effect further. Based on prior reports [68,69,70], the dosing used in our experiments is expected to achieve concentrations of ribavirin similar to those seen in the patients routinely treated with this agent for viral infections. There may be some room for dose escalation of ribavirin, as a higher risk of adverse side effects may be better justified in the context of treating an otherwise terminal metastatic disease. Another avenue for improvement may be through a combination with immune therapy. Despite promising early observations [71], attempts to combine MEK inhibition with immune checkpoint inhibition for the treatment of mismatch-proficient CRC so far have yielded underwhelming results in phase 3 clinical testing [72]. Our findings suggest that the efficacy of such a combination may be significantly increased by the addition of ribavirin (Figure 5). While our study explored only one drug combination and only one drug sequencing strategy (ICI lead-in before targeted therapy), it may be worth exploring in the future whether other forms of immunotherapy and other treatment sequences might further boost the anti-cancer effect. The immune microenvironment in the liver, the most common site of CRC metastasis, may present particular challenges to treatment [73]. Encouragingly, ribavirin was successfully used in combination with interferon to treat viral infection in the liver [74], suggesting that the drug has excellent bioavailability and can cooperate with an immunostimulatory intervention in that organ.

Importantly, while the drug combinations exceeded the efficacy of individual compounds in our studies, it remains to be elucidated whether they also surpass the efficacy of conventional chemotherapy, which, so far, remains the standard of care. Indeed, as is suggested by a recent report on combining encorafenib and cetuximab with oxaliplatin, leucovorin, and fluorouracil (mFOLFOX6) in BRAF-mutant CRC [16], the future use of the new drug combination may not be as an alternative, but as a companion to the conventional chemotherapy. In addition, even if the activity of the new combination will not be superior to that of the first-line standard of care, there is an urgent unmet need for additional lines of therapy for recurring disease in heavily pretreated patients. In this regard, the question of any possible cross-resistance between conventional chemotherapy and the targeted combinations still needs to be explored.

Direct therapeutic targeting of oncogenic RAS has recently become a reality, but the experience in the context of metastatic CRC suggests the need to increase its efficacy and to expand the range of targetable RAS variants. In this regard, it is interesting that a pan-RAS inhibitor synergizes with ribavirin in a cell line with KRAS^G12D^ mutation. We believe that the possible synergy between the new inhibitors of RAS and those of IMPDH, including in the context of immune therapy, merits further investigation.

The current study was focused on BRAF- and RAS-mutant variants of CRC. However, there is evidence [75] that hyperactivation of the MAPK cascade is an important mechanism used by BRAF and RAS wild-type CRC to circumvent the anti-EGFR therapy, which is commonly used in this variant of CRC. Furthermore, EGFR oncogenic signaling critically relies on RAC1 activation [76], and RAC1 activity was implicated in resistance to an EGFR inhibitor [77]. Therefore, we believe it would be interesting to evaluate whether concurrent treatment with a MEKi/IMPDHi combination may offer a benefit to this category of patients.

Finally, it is important to note that the pathways and their individual components targeted in the present report are important oncogenic drivers in a wide variety of malignancies. Therefore, we believe that the possibility of a synergy between IMPDH and MAPK cascade inhibitors in other cancers merits future investigation.

## 4. Materials and Methods

### 4.1. Cell Culture and Reagents

All cell lines were cultured in high-glucose DMEM containing penicillin (100 Units/mL), streptomycin (100 ug/mL), and 10% FBS. All cell lines were tested for mycoplasma using the MycoAlert Mycoplasma Detection Kit. The numbers of remaining cells in treated cultures were compared using the methylene blue staining and extraction method, as described previously [30].

Selumetinib was purchased from Selleckchem (S1008), MedChemExpress (HY-50706), and Adooq (10257-250). Cobimetinib was purchased from Adooq (A11441). PLX8394 was purchased from Adooq (A16840). Mirdametinib was purchased from Selleckchem (S1036). Binimetinib was purchased from Adooq (A11493). Ribavirin (RBV) was purchased from Selleckchem (S2504), MedChemExpress (HY-B0434), and Adooq (A10788). Mizoribine was purchased from Selleckchem (S1384) and Cayman Chemical (23128). Vemurafenib was purchased from Selleckchem (S1267) and LC-Laboratories (V-2800). Encorafenib was purchased from MedChemExpress (HY-15605). EHT1864 was purchased from Adooq (A13886). No differences in efficacy were noted whenever the same compound was procured from multiple vendors.

RNA interference using shRNA-expressing constructs was performed as before [33,63].

### 4.2. Cell Number Comparison in Treated Cultures

Compounds were added to sub-confluent cultures of cells that were plated the night before. Following the desired length of treatment, the numbers of remaining cells were compared by the methylene blue staining and extraction method as described in [78] using a PerkinElmer Victor X3 Multimode Plate Reader. Background-corrected values for each treatment condition were normalized to the average values obtained in parallel cultures of cells treated with a corresponding vehicle control. Every treatment condition in every survival experiment was investigated in four biological replicas.

### 4.3. Western Blotting and Antibodies

Cells were lysed in 1 × RIPA Lysis Buffer (EMD Millipore) supplemented with PhosSTOP Phosphatase Inhibitor and Complete Protease Inhibitor cocktails (Roche). pERK (sc-7383), total ERK (sc-514302), HRP-conjugated β-actin (sc-47778) primary antibodies, and mouse anti-rabbit IgG HRP secondary antibody (sc-2357) were all purchased from Santa Cruz Biotechnology. All prepared protein samples were separated on Mini-PROTEAN TGX gels (Bio-Rad) in denaturing conditions. Following protein transfer onto PVDF membranes (Thermo Scientific), the membranes were blocked in either 5% Blotting Grade Blocker Non-Fat Dry Milk (Bio-Rad) dissolved in Tris-Borate-SDS-Tween 20 (TBST) or 5% BSA-TBST per the recommended probing conditions for each antibody, followed by overnight probing at 4 °C. Following incubation with primary antibody, membranes were washed in TBST and incubated in secondary antibody diluted in 5% Blotting Grade Blocker Non-Fat Dry Milk-TBST for 1 h at room temperature on a shaker. Immunobilon Classico (EMD Millipore) and SuperSignal West Pico PLUS Chemiluminescent Substrate (Thermo Scientific) were used for developing membranes for chemiluminescent imaging. Proteins were imaged on a Bio-Rad ChemiDoc Touch Imaging System (software version 1.2.0.12) and quantified using Fiji software (ImageJ version 1.54P).

### 4.4. Rac1 Activity Assay

Rac1 activity was measured in protein lysates collected from cell lines treated under various conditions using an Active GTPase Kit (1860S) purchased from Cell Signaling Technology. Lysates were prepared per the kit’s instructions (the 1 × Lysis/Binding/Wash buffer was supplemented with Complete Protease Inhibitor Cocktail). Samples containing immunoprecipitated active-Rac1 and the corresponding input samples were immunoblotted for Rac1 using the primary antibody supplied with the kit. The signals were acquired using the Bio-Rad ChemiDoc Touch Imaging System and quantified using Fiji software (ImageJ version 1.54P).

### 4.5. Comparison of Intracellular GTP Content

Comparison of intracellular GTP content was performed as previously described [36]. Briefly, approximately five million cells were collected by trypsinization after the indicated treatments. The cells were lysed in 0.4 N perchloric acid, followed by neutralization to pH 8 with potassium hydroxide. NTPs were separated on a strong anion exchange reverse phase column (Millipore Sigma, Cat # 50193-U) using a gradient high-performance liquid chromatography system (Agilent 1100, Santa Clara, CA, USA) equipped with a photodiode array detector and controlled by the Agilent ChemStation B.04.03-SP1 software. NTPs were identified by their UV absorbance spectrum and quantified by comparing the integrated area of their absorbance peaks to that of known NTP standards.

### 4.6. Treatment of Xenografts in Mice

All animal work was performed at the Comparative Oncology Shared Resource of the Roswell Park Comprehensive Cancer Center and in accordance with the protocol approved by the Institutional Animal Care and Use Committee of Roswell Park Comprehensive Cancer Center.

HCT15 and RKO xenografts were established by injecting 1 × 10^6^ cells into the right flanks of 6- to 8-week-old female SCID animals subcutaneously. Tumor volumes were measured as described before [79]. The tumors were allowed to grow until they reached approximately 100 mm^3^, at which time the animals were randomly assigned to each treatment group and started to receive oral gavage daily. The mice bearing HCT15 tumors received daily either 30 mg/kg selumetinib, 20 mg/kg ribavirin, the combination thereof, or the vehicle control (25% DMSO/30% PEG-300/45% molecular grade water). The mice bearing RKO tumors received either daily 5 mg/kg cobimetinib, 20 mg/kg ribavirin, the combination thereof, or the vehicle control (25% DMSO/30% PEG-300/45% molecular grade water). During the experiment, the mice were sacrificed when they became moribund or if the tumor size exceeded 2 cm^3^.

For immunotherapy experiments, 2.5 × 10^5^ CT26 cells were subcutaneously injected into the right flanks of 6- to 8-week-old female Balb/c mice. Tumors were allowed to grow until they reached 50 mm^3^–100 mm^3^ in volume before the animals were randomly assigned to receive 200 ug of Anti-PDL1 antibody or IgG control (Bio X Cell). Animals were treated with three 3-day cycles of either 200 ug anti-PDL1 immune checkpoint blockade or 200 ug IgG isotype via intraperitoneal injection on days 1, 4, and 7. On day 10, animals from each treatment arm were further randomly assigned to receive either daily 30 mg/kg selumetinib, 20 mg/kg RBV, the combination thereof, or the vehicle control (25% DMSO/30% PEG-300/45% molecular grade water) via oral gavage. During the experiment, the animals were sacrificed upon becoming moribund or when tumors reached 2 cm^3^.

All control and drug-treated animals were included in the final survival analysis.

### 4.7. Statistical Analysis

For cell survival assays, drug synergy scores were calculated according to the Loewe synergy model using SynergyFinder (version# 16.01.2024-R-3.8.2-dev) [80]. This software calculates the mean deviation from Loewe additivity [81] across the entire range of presented experimental conditions, along with the corresponding *p*-value, which assumes the absence of synergy as the null-hypothesis. It also plots the estimated synergy scores across the entire range of experimental conditions in the form of a three-dimensional diagram, where the higher synergy scores are emphasized by a more intense color for improved visualization. Other indicated statistical calculations, including the generation and analysis of Kaplan–Meier survival plots, were performed using GraphPad Prism 10 (GraphPad Software, LLC, Boston, MA, USA). *p*-values below 0.05 were used as the indication of statistical significance.

## Data Availability

The raw data supporting the conclusions of this article will be made available by the authors on request.

## Supplementary Materials

The following supporting information can be downloaded at: https://www.mdpi.com/article/10.3390/ijms262411959/s1.

## Abbreviations

The following abbreviations are used in this manuscript:

BIN	Binimetinib
BRAFi	Inhibitor of B-Raf proto-oncogene
COB	Cobimetinib
CRC	Colorectal cancer
DMSO	Dimethyl sulfoxide
EHT	EHT1864
ENC	Encorafenib
ICI	Immune checkpoint inhibitor
IMPDHi	Inhibitor of inosine monophosphate dehydrogenase (IMPDH)
MAPK	Mitogen-activated protein kinase
MEKi	Inhibitor of mitogen-activated protein kinase kinase (MEK)
MIR	Mirdametinib
MIZ	Mizoribine
MMR	Mismatch repair
RBV	Ribavirin
SEL	Selumetinib
VEM	Vemurafenib
