# Supplementary material for: Inhibitors of De Novo Guanylate Biosynthesis Enhance the Potency of MAPK Cascade Inhibitors Against Colorectal Cancer
Source: Int J Mol Sci. 2025 Dec 11;26(24):11959. doi: 10.3390/ijms262411959 (PMC12732750; doi:10.3390/ijms262411959)
Supplement: Supplementary file 1 [file ijms-26-11959-s001.zip › ijms-3979487-supplementary.pdf]

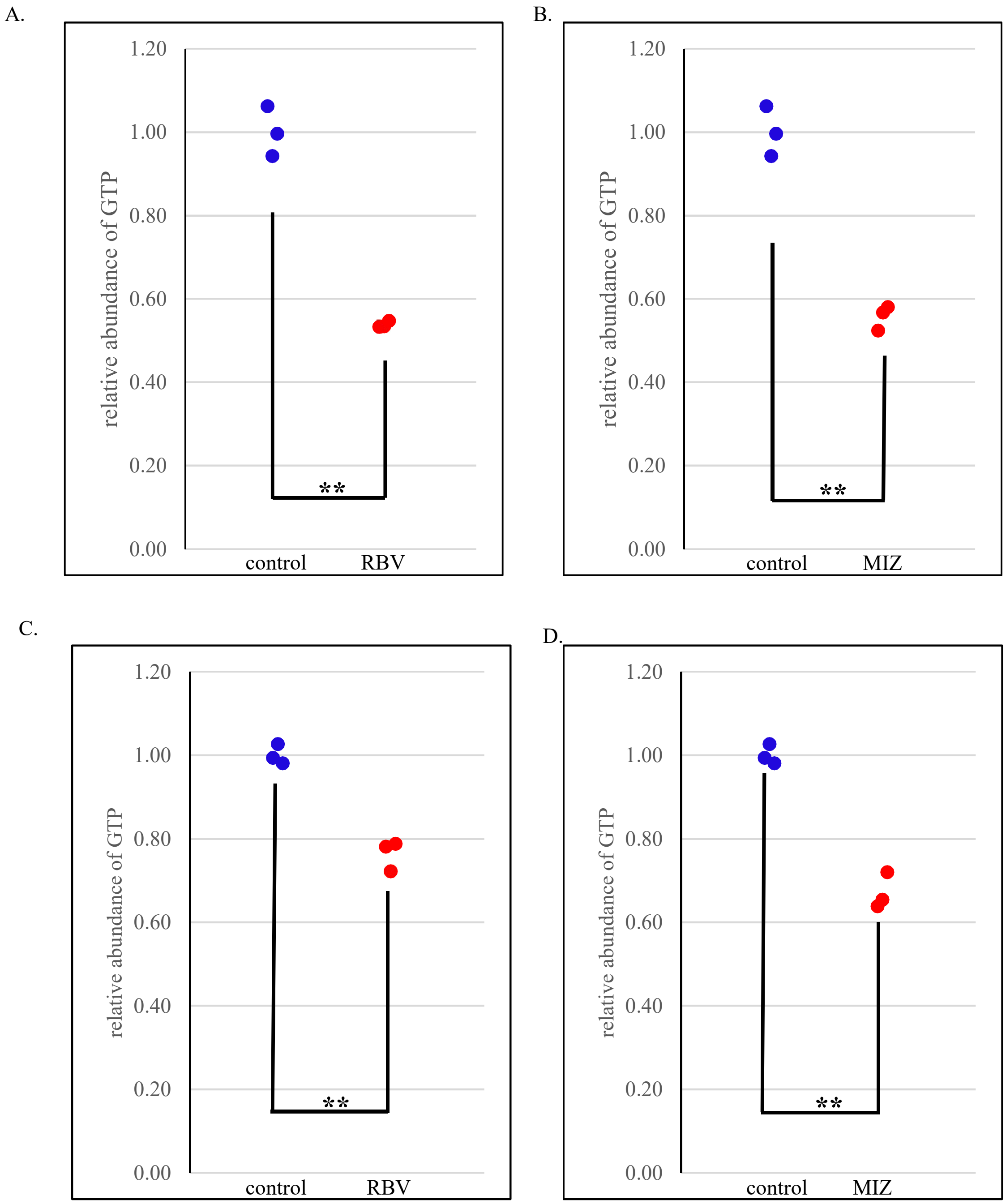

Figure S1. IMPDH inhibitors reduce the abundance of GTP in colorectal cancer cell lines. A. The abundance of GTP in HCT15 cells treated for 72h with 10µM ribavirin ("RBV") was measured as described in Materials and Methods and plotted relative to that in a parallel untreated control culture. The results of three independent experiments are shown. "**"-p≤0.005. B. The abundance of GTP in HCT15 cells treated for 72h with 10µM Mizoribine ("MIZ") was analyzed as in A. C. The abundance of GTP in RKO cells treated for 72h with 10µM ribavirin was analyzed as in A. D. The abundance of GTP in RKO cells treated for 72h with 10µM mizoribine was analyzed as in A.

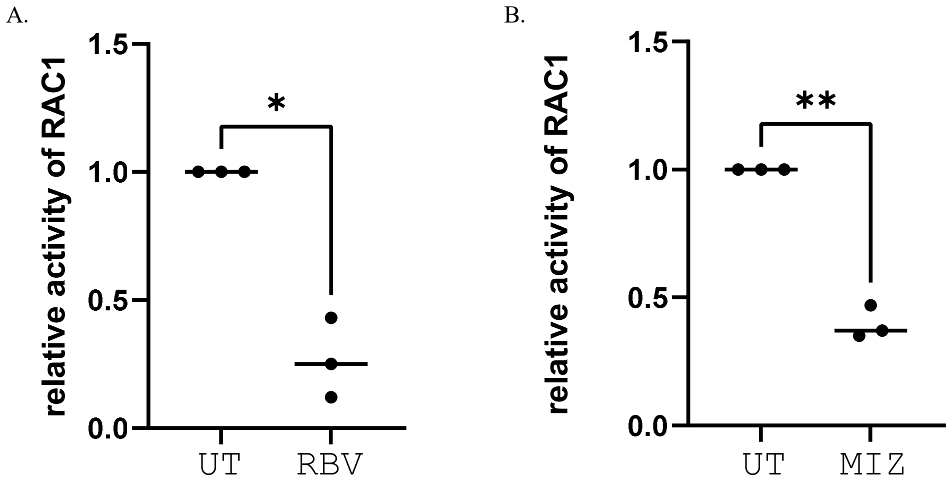

Figure S2. Treatment with inhibitors of guanylate biosynthesis causes a decrease in the levels of active RAC1 in HCT15 cells. A. The amounts of active (capable of binding to PAK1) and total RAC1 were determined using the Active GTPase Kit (Cell Signaling Technology) in the lysates of HCT15 cells, untreated ("UT") or treated for 72 hours with 10μM ribavirin ("RBV"). The signals were acquired using Bio-Rad ChemiDoc Touch Imaging System and quantified using Fiji software (ImageJ version 1.54P). The amount of active RAC1 in a lysate was normalized to that of the total RAC1 protein and is shown relatively to the value in the parallel untreated culture in the same experiment. The values from three independent experiments are shown. "*" – $p < 0.05$ (paired Student's t-test). **B.** Relative activity of RAC1 in HCT15 cells, untreated ("UT") or treated for 72 with 10μM mizoribine ("MIZ"), was determined and analyzed as in A. The values from three independent experiments are shown. "**" – $p < 0.005$ (paired Student's t-test).

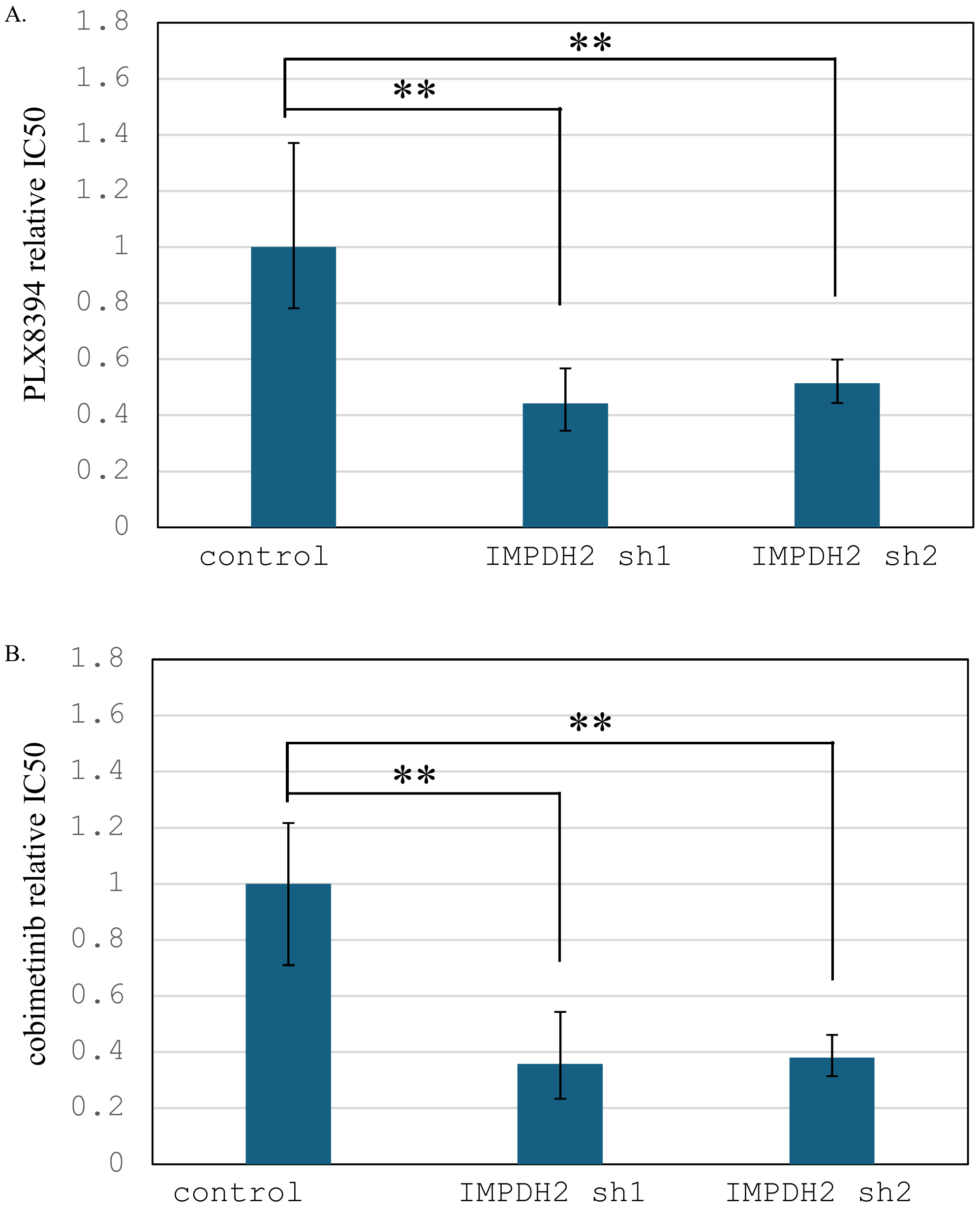

Figure S3. Interference with IMPDH2 reduces the sensitivity of RKO cells to inhibitors of the MAPK cascade. A. RKO cells harboring IMPDH shRNAs or the respective control vector were treated for 5 days with a 1-160 nM range of PLX8394 (BRAF inhibitor) and the numbers of the remaining cells were compared using the methylene blue staining and extraction method. 50% inhibitory concentrations (IC50) were calculated and compared using GraphPad Prizm 9. The values are shown relative to that in the control cells. Error bars -95% confidence intervals. "**" – $p<0.005$. B. IC50 values after a 5-day treatment with 0-48 nM cobimetinib (MEK inhibitor) were determined and compared as in A.
